# Arginine Metabolism by Macrophages Promotes Cardiac and Muscle Fibrosis in *mdx* Muscular Dystrophy

**DOI:** 10.1371/journal.pone.0010763

**Published:** 2010-05-21

**Authors:** Michelle Wehling-Henricks, Maria C. Jordan, Tomomi Gotoh, Wayne W. Grody, Kenneth P. Roos, James G. Tidball

**Affiliations:** 1 Department of Integrative Biology and Physiology, University of California Los Angeles, Los Angeles, California, United States of America; 2 Cardiovascular Research Laboratory, Department of Physiology, David Geffen School of Medicine, University of California Los Angeles, Los Angeles, California, United States of America; 3 Department of Molecular Genetics, Kumamoto University School of Medicine, Kumamoto, Japan; 4 Department of Human Genetics, David Geffen School of Medicine, University of California Los Angeles, Los Angeles, California, United States of America; 5 Department of Pathology and Laboratory Medicine, David Geffen School of Medicine, University of California Los Angeles, Los Angeles, California, United States of America; 6 Department of Pediatrics, David Geffen School of Medicine, University of California Los Angeles, Los Angeles, California, United States of America; 7 Molecular, Cellular and Integrative Physiology Program, University of California Los Angeles, Los Angeles, California, United States of America; Hospital Vall d'Hebron, Spain

## Abstract

**Background:**

Duchenne muscular dystrophy (DMD) is the most common, lethal disease of childhood. One of 3500 new-born males suffers from this universally-lethal disease. Other than the use of corticosteroids, little is available to affect the relentless progress of the disease, leading many families to use dietary supplements in hopes of reducing the progression or severity of muscle wasting. Arginine is commonly used as a dietary supplement and its use has been reported to have beneficial effects following short-term administration to mdx mice, a genetic model of DMD. However, the long-term effects of arginine supplementation are unknown. This lack of knowledge about the long-term effects of increased arginine metabolism is important because elevated arginine metabolism can increase tissue fibrosis, and increased fibrosis of skeletal muscles and the heart is an important and potentially life-threatening feature of DMD.

**Methodology:**

We use both genetic and nutritional manipulations to test whether changes in arginase metabolism promote fibrosis and increase pathology in mdx mice. Our findings show that fibrotic lesions in mdx muscle are enriched with arginase-2-expressing macrophages and that muscle macrophages stimulated with cytokines that activate the M2 phenotype show elevated arginase activity and expression. We generated a line of arginase-2-null mutant mdx mice and found that the mutation reduced fibrosis in muscles of 18-month-old mdx mice, and reduced kyphosis that is attributable to muscle fibrosis. We also observed that dietary supplementation with arginine for 17-months increased mdx muscle fibrosis. In contrast, arginine-2 mutation did not reduce cardiac fibrosis or affect cardiac function assessed by echocardiography, although 17-months of dietary supplementation with arginine increased cardiac fibrosis. Long-term arginine treatments did not decrease matrix metalloproteinase-2 or -9 or increase the expression of utrophin, which have been reported as beneficial effects of short-term treatments.

**Conclusions/Significance:**

Our findings demonstrate that arginine metabolism by arginase promotes fibrosis of muscle in muscular dystrophy and contributes to kyphosis. Our findings also show that long-term, dietary supplementation with arginine exacerbates fibrosis of dystrophic heart and muscles. Thus, commonly-practiced dietary supplementation with arginine by DMD patients has potential risk for increasing pathology when performed for long periods, despite reports of benefits acquired with short-term supplementation.

## Introduction

Fibrosis is a prominent feature of Duchenne muscular dystrophy (DMD) that underlies many aspects of the disease that lead to death. Respiratory insufficiency, the leading cause of death among DMD patients, results from progressive fibrosis that diminishes the contractile function of the respiratory muscles [1]–[3]. Respiratory function is further compromised by thoracic deformities caused by fibrosis of postural muscles [4]–[6]. Myocardial fibrosis, the second leading cause of death in DMD [3], [7], [8], occurs in more than 96% of DMD hearts and causes cardiac dysfunction that leads to heart failure. Furthermore, fibrotic lesions in the myocardium can act as foci of ventricular arrhythmias that are common and often fatal in DMD patients [7], [9], [10]. Additionally, fibrosis of limb muscles causes permanent, immobilizing contractures that impede ambulation [11]. Despite the severe effects of fibrosis, little is known about the mechanisms that induce the deposition of connective tissue in dystrophin-deficient muscle and heart.

The primary cause of DMD is a mutation of the dystrophin gene that results in loss of dystrophin protein [12]. Dystrophin is a member of a transmembrane complex of structural and signaling proteins, called the dystrophin glycoprotein complex (DGC). Dystrophin-deficiency causes great reductions in DGC proteins at the sarcolemma [13] and this increases the membrane's susceptibility to mechanical damage and compromises functions related to the loss of signaling proteins in the DGC [14]–[16].

Neuronal nitric oxide synthase (nNOS) is a member of the DGC whose loss from dystrophic muscle plays a significant role in the disease [17], [18]. Because arginine metabolism by nNOS yields production of nitric oxide (NO), a versatile and physiologically-important signaling molecule, nNOS-deficiency produces numerous defects in muscle homeostasis. Several investigations have been directed toward identifying features of DMD pathology that are primarily attributable to nNOS-deficiency by analyzing the effect of expressing a muscle-specific nNOS transgene in the *mdx* mouse model of DMD. In the nNOS transgenic *mdx* mice used for those analyses, NO production by the muscles was returned to wild-type levels [15]. Among the improvements observed, skeletal muscles and hearts from *mdx* mice experienced large, significant reductions in inflammation that were accompanied by reductions in skeletal muscle fibrosis [our unpublished data] and complete prevention of myocardial fibrosis that was attributable to nNOS transgene expression [16]. However, whether those reductions in fibrosis resulted from reductions in inflammation or some other NO-mediated process could not be addressed by the findings.

The abilities of macrophages to drive tissue fibrosis and of NO to function as an anti-inflammatory molecule support the hypothesis that the reductions in *mdx* fibrosis that were achieved by normalizing muscle NO production could be secondary to an anti-inflammatory, NO-mediated effect. Recent findings support this possibility by showing that *mdx* muscle is infiltrated by pro-fibrotic M2a macrophages [19]. M2a macrophages express high levels of arginase that metabolizes arginine to produce pro-fibrotic agents such as polyamines and ornithine [20]–[23]. During the early, acute stage of *mdx* pathology, *mdx* muscles are also invaded by M1 macrophages that express high concentrations of inducible nitric oxide synthase (iNOS). Arginine metabolism by iNOS in M1 macrophages produces toxic levels of NO that lyse muscle cell membranes [19]. *Mdx* muscle is subsequently invaded by M2c macrophages that do not express iNOS, but release IL-10 that can inactivate the M1 phenotype [19], [24]. Both M1 and M2a populations are present during the acute, necrotic phase of the *mdx* pathology where they compete for arginine, the common substrate for iNOS and arginase [19]. However, deactivation of the M1 phenotype by M2c macrophages can increase substrate availability for arginase [19]. The shift of arginine metabolism from iNOS to arginase, called the “arginine switch,” can create a more pro-fibrotic environment [25].

Macrophage-driven fibrosis could be further exacerbated by nNOS deficiency in *mdx* tissues because arginine oxidation by NOS yields metabolites that inhibit arginase and that would be lost in dystrophin-deficient muscles [26], [27]. Nitrite, which is a stable oxidation product of NO, also reduces arginase activity in vitro [28]. In addition, NO can S-nitrosylate an active site cysteine on ornithine decarboxylase (ODC), thereby inactivating the enzyme [29]. This S-nitrosylation prevents ODC from converting ornithine to polyamines that can promote proliferation of fibroblasts involved in connective tissue production [29]. Collectively, these findings show that NOS is a powerful negative regulator of arginase, and the ability of arginase-derived metabolites to promote fibrosis could be magnified by the loss of nNOS from dystrophic muscle.

Tissue fibrosis may also be increased by dietary supplementation with arginine, increasing substrate availability for arginase. For example, dietary supplementation with arginine for 2 weeks in humans increased connective tissue deposition in wounds [30]. We believe that dietary arginine supplementation for DMD patients would have an even greater effect on increasing fibrosis because of the lack of competition for arginine by nNOS. Unfortunately, dietary, arginine-supplementation is a common practice for DMD patients. Arginine supplementation by DMD patients stems from reports that short-term, arginine treatment for 2–6 weeks has beneficial effects in young, *mdx* mice that include increased utrophin expression, increased force production, decreased inflammation and decreased fiber damage [31]–[34]. However, the effects of long-term, dietary, arginine supplementation in the treatment of dystrophinopathy are unknown and have the unexplored potential to promote tissue fibrosis.

In this study, we tested whether the loss of nNOS in nNOS-null mutant mice or the displacement of nNOS from the sarcolemma of muscle cells in α-syntrophin null mice was sufficient to increase fibrosis or cause functional defects in the heart. We then assessed whether macrophages that express arginase-2 are present in the fibrotic lesions of *mdx* muscle, and whether their arginase expression is modulated by cytokines that promote the M2 phenotype. We then investigated whether ablation of arginase-2 expression in *mdx* mice affects fibrosis of skeletal muscle or the myocardium or influences defects in cardiac function in *mdx* mice. Conversely, increasing arginase substrate availability by dietary supplementation with arginine was analyzed to assay for effects on *mdx* pathology. Collectively, our findings reveal a new, profibrotic pathway that plays an important role in the pathophysiology of muscular dystrophy, and provide a caution for the use of long-term dietary supplementation with arginine for DMD patients.

## Materials and Methods

### Ethics statement

Experiments were conducted according to the National Institutes of Health Guide for the Care and Use of Laboratory Animals and the UCLA Institutional Animal Care and Use Committee. The experimental protocols used in this investigation were approved by the UCLA Animal Research Committee (protocol #ARC-2004-216). UCLA is accredited by the Association for Assessment and Accreditation of Laboratory Animal Care (#A3196-01).

### Animals

Mice were obtained from our breeding colony at the UCLA vivarium. C57BL/6, *mdx* and nNOS -/- mice breeding pairs were purchased from The Jackson Laboratory (Bar Harbor, ME, USA). Mice that were null mutants for arginase-2 (A2ko null mice) were provided by Dr. William O'Brien (Baylor College of Medicine, Houston, TX, USA) and crossed onto the *mdx* background in our lab. Mice that were null mutants for α-sarcoglycan (α-sko) mice were provided by Dr. Stanley Froehner and Dr. Marvin Adams (University of Washington, Seattle, WA, USA). C57BL/6 mice were used for wild-type controls.

Only male mice were used in experimentation to avoid effects of gender dimorphism on fibrosis [35]. Analyses were performed on 18-month-old mice, during late-stage *mdx* fibrosis. However, nNOS null mice were analyzed at 12 months of age, which nears their near-maximum lifespan due to gastric complications caused by nNOS deficiency [36].

### Th2-stimulation of *mdx* muscle macrophages

Macrophages were isolated from *mdx* skeletal muscle as previously described [15]. Muscle macrophages were resuspended in Dulbecco's modified eagle medium (DMEM) (Sigma, St. Louis, MO, USA) containing 10% fetal bovine serum (Omega Scientific, Tarzana, CA, USA) and plated at 1×10^6^ cells/ml. Cultures were stimulated for 24 hours with either 10 ng/ml IL-4), 10 ng/ml IL-10, 20 ng/ml IL-13 or no cytokine as controls (all cytokines, BD Biosciences, San Jose, CA, USA. A minimum of five replicates per condition was assayed at each age.

### Arginase activity assay

Arginase activity was determined according to Corraliza et al [37]. Cells were lysed with 0.2% Triton-100 (EMD Biochemicals, Gibbstown, NJ, USA) containing a protease inhibitor cocktail (Sigma). Cells were scraped into 10 mM MnCl_2_, 50 mM Tris-HCl, pH 7.5 and heated to 56°C for 10 minutes to activate arginase. Substrate hydrolysis was performed by adding 0.5 M arginine, pH 9.7 to the cell lysate followed by a 1-hour incubation at 37°C. The reaction was stopped by adding H_2_SO_4_ and H_3_PO_4_. Samples were heated to 100°C for 45 minutes after adding α-isonitrosopropriophenone and urea content was then measured spectrophotometrically at 540 nm. Values were normalized to protein content of the cell lysate. A minimum of five replicates per condition was assayed at each age.

### Arginine treatment

*Mdx* and C57BL/6 mice received drinking water supplemented with 5 mg/ml of L- or D-arginine (EMD Biochemicals, San Diego, CA) from 3 weeks to 18 months of age. This quantity of L-arginine supplementation has been reported to decrease muscle pathology in young, *mdx* mice and increase connective tissue deposition during wound healing [30], [38]. Water intake was measured weekly and was similar between L- and D-arginine-treated groups. Each treatment group consisted of 10 animals. D-arginine is used in control treatments because it is a non-metabolized, stereoisomer of L-arginine. This control differs from previous investigations of the short-term effects of L-arginine treatments on *mdx* pathology, which used saline [31]–[34].

### Histology

Dissected tissues were stored in isopentane at −80°C until they were sectioned at a thickness of 10 µm. Sections used for immunostaining were fixed in acetone and endogenous peroxidase activity was quenched with 0.03% hydrogen peroxide. Non-specific binding was blocked with phosphate buffered saline (PBS) containing 2% gelatin and 3% bovine serum albumin. Tissues were incubated with primary antibodies for 3 hours followed by a biotinylated, second antibody and horseradish peroxidase-avidin D (Vector Laboratories, Burlingame, CA, USA). Signals were visualized using 3-amino-9-ethyl carbazole (AEC, red) (Vector) as substrate. Primary antibodies used were monoclonal rat anti-mouse F4/80 for macrophages and rat anti-mouse CD4 purified from supernatants of hybridoma cultures (hybridomas from American Type Culture Collection, Bethesda, MD, USA), monoclonal rat anti-mouse CD8 (Southern Biotech, Birmingham, AL, USA), monoclonal rat anti-mouse Ly-6G for neutrophils (Serotec, Raleigh, NC, USA) and polyclonal rabbit anti-murine eosinophil granule major basic protein (a gift from Dr. J.J. Lee, Mayo Clinic Scottsdale, AZ, USA) [39]. The concentrations of inflammatory cells were measured by histomorphometry as previously described [40]. Quantitative histological analyses were performed on seven animals per genotype or treatment group.

Collagen staining was performed as described above, but with the following modifications. The sections were not quenched with hydrogen peroxide, and following a 3-hour incubation with primary antibody the sections were incubated with host-appropriate fluorescent-conjugated secondary antibodies (Vector) for 1 hour. Primary antibodies used were rabbit anti-rat collagen type I (Chemicon, Temecula, CA, USA), rabbit anti-rat collagen type III (Chemicon), goat anti-human collagen type IV (Southern Biotech), and goat anti-human collagen type V (Southern Biotech).

### Hydroxyproline assay

Skeletal muscle (quadriceps, soleus, diaphragm and longissimus dorsi) and cardiac fibrosis was determined by quantifying hydroxyproline in tissues from 5 animals per group according to the technique of Kivirikko et al. [41]. Hydroxyproline is present almost exclusively in collagens, so that its quantification provides an accurate assessment of the relative quantity of collagen in a tissue.

### Kyphotic index

Kyphosis was measured using the murine kyphotic index (KI) as established by Laws and Hoey [5]. KI was measured from a radiograph of a mouse in lateral recumbancy by drawing a line (AB) between the posterior most aspects of C6 and L6 and a line (CD) from AB to the point of greatest dorsal curvature of the spine. KI is calculated as AB/CD. KI was measured in at least 6 animals from each group, as indicated in the text.

### Echocardiography

Two-dimensional, M-mode echocardiography and spectral Doppler images were obtained to analyze cardiac function, left ventricular chamber size, wall thickness, ventricular and valve function and blood flow [42]. At least 5 mice per group were sedated with isoflurane vaporized in oxygen, maintained at physiological heart rates (500–600 bpm) and imaged using a Siemens Acuson Sequoia C256 equipped with a 15L8 15 MHz probe (Siemens Medical Solutions, Mountain View, CA). Data were analyzed using the Acuson and AccessPoint software (Freeland Systems LLC, Santa Fe, NM, USA).

### Western blot analysis

Tibialis anterior muscles for western analysis were stored in liquid nitrogen until homogenized in 40 volumes sodium dodecyl-sulfate-polyacrylamide gel electrophoresis (SDS-PAGE) reducing buffer (80 mM Tris-HCl pH 6.8, 0.1 M dithiothreitol, 70 mM SDS and 1.0 mM glycerol) with protease inhibitor cocktail (Sigma). Cultured macrophages were rinsed once with PBS before collecting in SDS-PAGE reducing sample buffer with protease inhibitor cocktail. All samples were heated to 100°C for 1 minute and then centrifuged for 1 minute at 12,000 g. Protein concentration of the supernatant was measured at 280 nm and samples were separated on 5% (for utrophin) or 12% (for arginase) SDS-PAGE gels. Proteins were transferred electrophoretically to nitrocellulose membranes and the efficiency and uniformity of loading was confirmed by staining membranes with 0.1% Ponceau S (Sigma). Membranes were incubated in blocking buffer (0.5% Tween-20, 0.2% gelatin and 3.0% nonfat dry milk) for 1 hour. Blots were probed with rabbit anti-arginase-1/2 [43], rabbit anti-utrophin (Novocastra, Bonnockburn, IL, USA) or mouse anti-nNOS (BD Transduction Labs, San Jose, CA, USA) for 3 hours followed by an anti-rabbit, HRP-conjugated antibody (Amersham, Buckinghamshire, UK). Washes in PBS with 0.1% Tween followed each incubation. Bands were visualized via enhanced chemiluminescence and quantified using Alpha Innotech imaging software (Alpha Innotech, San Leandro, CA, USA). Each western blot was then subsequently reprobed with a rabbit anti-skeletal alpha-actin antibody (Sigma) to confirm that loading and protein transfer were consistent between lanes.

### Zymography

Gelatinase zymography was performed using a modification of Kherif et al [44]. Frozen tibialis anterior muscles were homogenized in 40 volumes of sample buffer (125 mM Tris, pH, 6.8, 20% glycerol, 4% SDS). After centrifuging the homogenates at 12,000 g and 4°C for 5 minutes, protein concentration of the supernatant was measured at 280 nm. We loaded 40 µg of each sample onto 10% acrylamide gels impregnated with 1 mg/ml gelatin and electrophoresed the samples at 4°C. The gels were agitated in renaturing buffer (2.5% Triton-X100) for 1 hour with 3 buffer changes. Gels were switched to developing buffer, pH 7.5 (50 mM Tris, 0.2 M NaCl, 5 mM CaCl2, 0.02% Brij-35) for two, 30-minute washes at room temperature and 24 hours at 37°C. Gels were stained overnight in Coomassie Blue R250 (Sigma) and clear bands indicating matrix metalloproteinase activity were visualized by destaining in 40% MeOH with 10% acetic acid. Bands were quantified using Alpha Innotech imaging software.

### Statistics

Statistical analyses were calculated using the one-way ANOVA or the non-parametric, Kruskal-Wallis test. The p value was set at p<0.05. Significant differences between groups were identified using Tukey's Post Hoc test.

## Results

### Disruption of nNOS expression or localization in the absence of inflammation is not sufficient to induce skeletal muscle or cardiac fibrosis

**Figure 1:**
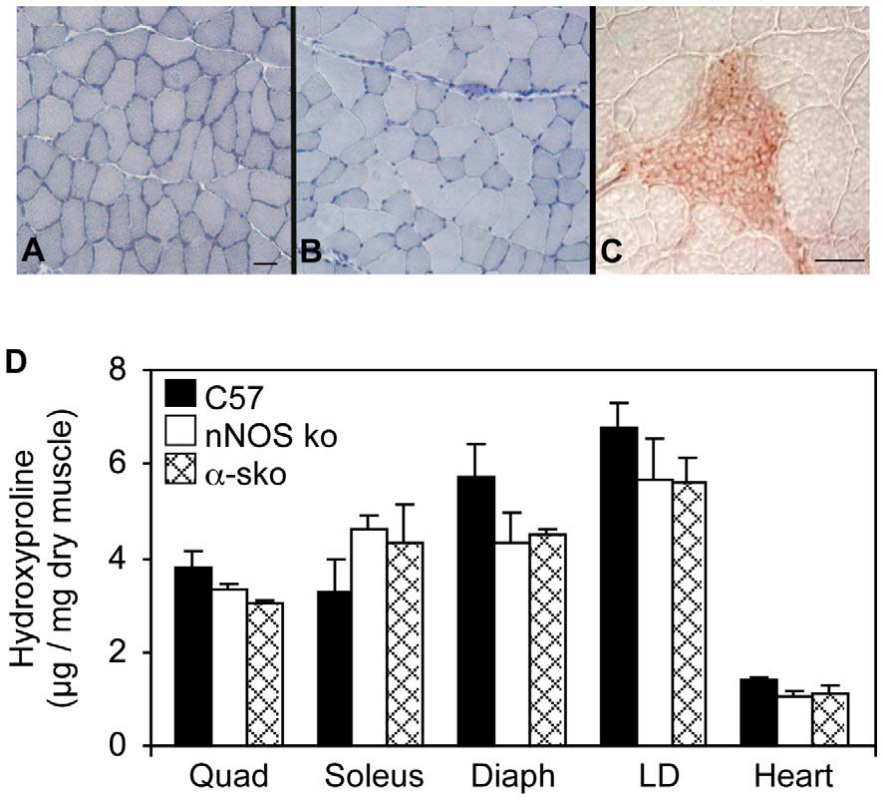
Mice with disrupted nNOS expression or localization do not exhibit inflammation or develop fibrosis. (A and B) Cross-sections of 12-month old nNOS null (A) and α−sko (B) quadriceps stained with hematoxylin are free of inflammation. Bar = 50 µm. (C) Mononuclear cells in an inflammatory lesion of 18-month-old *mdx* quadriceps stain positive for arginase expression. Bar = 50 µm. (D) Mice lacking nNOS expression (nNOS ko) or localization to the sarcolemma (α-sko) do not develop pathological fibrosis in quadriceps (Quad), soleus, diaphragm (Diaph), longissimus dorsi (LD) and heart tissues. C57 mice (n = 5) and α-sko mice (n = 5) were 18-months old. nNOS ko mice (n = 5) were 12-months old.

We tested whether fibrosis could result from disruptions in nNOS expression or localization because nNOS expression is greatly reduced in the hearts and muscles of *mdx* mice and previous investigations showed that nNOS transgene expression in *mdx* muscles reduces muscle fibrosis and prevents cardiac fibrosis [16]. We examined nNOS null mice [36] and α−sko mice in which nNOS is displaced from the muscle cell membrane to the cytosol [45], [46], but do not experience chronic inflammation or myopathy. Examination of skeletal muscles and hearts from nNOS null and α−sko mice confirmed the absence of inflammation in both models (Fig. 1A and B). Quantification of connective tissue in skeletal muscles and hearts showed that neither the nNOS null nor the α−sko mice developed fibrosis (Fig. 1D). Thus, neither the absence of nNOS or its mislocalization is sufficient to induce the fibrosis present in aged *mdx* muscles and hearts. Additionally, nNOS null and α−sko mice were less kyphotic than the *mdx* mice (kyphotic index (KI): *mdx* = 2.9 (sem = 0.04), n = 10; nNOS null = 3.3 (sem = 0.2), n = 7; α−sko = 3.7 (sem = 0.08), n = 6) showing that kyphosis that results from muscle fibrosis is independent of reduced NOS expression or changes in nNOS distribution.

### Disruption of nNOS expression or localization is not sufficient to yield defects in cardiac structure and function that are characteristic of *mdx* dilated cardiomyopathy

Previous work has shown that *mdx* mice that are about 10 months of age exhibit morphological features of dilated cardiomyopathy (DCM) that are detectable by echocardiography, and noted the association between the presence of myocardial fibrosis and inflammation with the occurrence of DCM in *mdx* mice [47]–[49]. Because nNOS-derived NO can contribute to vasodilation under some conditions [50] and reductions in NO can lead to increased peripheral resistance that can subsequently promote DCM [51], we assayed whether the genetic ablation of nNOS was sufficient to induce features of DCM that were detectable by echocardiography. However, all aspects of nNOS null cardiac structure and function that were examined by echocardiography did not differ significantly from wild-type hearts (Table 1), showing that loss of nNOS in the absence of inflammation and fibrosis is insufficient to cause defects that are characteristic of DCM in the dystrophic heart. Similarly, normal cardiac structure and function were observed in α−sko mice for all ECG parameters that were examined, which indicated that loss of nNOS from the cell membrane is insufficient to induce defects in cardiac function that resemble DCM (Table 1).

**Table 1:** Echocardiographic evaluation of mice with modified arginase-2 or nNOS expression.

	VST (mm)	EDD (mm)	PWT (mm)	FS (%)	Vcf (mm/s)	LvEF (%)
C57	0.69 (0.02)	4.16 (0.11)	0.70 (0.02)	29.6 (2.54)	5.56 (0.57)	62.6 (3.51)
A2ko	0.72 (0.03)	4.22 (0.10)	0.76 (0.01)	31.8 (1.50)	6.12 (0.40)	66.8 (2.30)
mdx	0.73 (0.01)	4.22 (0.22)	0.65 (0.004)	26.5 (2.60)	5.83 (0.60)	58.2 (5.20)
A2ko/mdx	0.76 (0.02)	4.70 (0.15)	0.73 (0.02)	23.9 (1.07)	5.31 (0.39)	53.6 (2.13)
α-sko	0.75 (0.06)	3.98 (0.12)	0.71 (0.04)	30.2 (2.40)	6.13 (0.46)	63.6 (3.84)
nNOS ko	0.74 (0.04)	4.43 (0.11)	0.74 (0.02)	23.5 (1.82)	4.23 (0.36)	53.7 (3.01)

Values are means with standard errors. VST  =  ventricular septal thickness; EDD  =  end diastolic diameter;

PWT  =  posterior wall thickness; FS  =  fractional shortening; Vcf  =  mean circumferential shortening rate; LvEF  =

left ventricular ejection fraction. Sample sizes: C57, n = 7; A2ko, n = 5; *mdx*, n = 5; A2ko/*mdx*, n = 6; α−sko, n = 5;

nNOS ko, n = 7.

The increase in end diastolic diameter (EDD) and decrease in fractional shortening (FS) in 10-month-old *mdx* hearts compared to control [47]–[49] is consistent with DCM in *mdx* mice at that age. Because fibrosis is increased in *mdx* hearts between 12 and 24 months of age [52], we assayed whether aging exacerbated defects in EDD or FS in *mdx* mice (Table 1). However, we observed no difference in either EDD or FS in the hearts of 18-month-old *mdx* mice compared to wild-type controls. Similarly, Cohn et al. [52] previously observed no difference in EDD in the hearts of *mdx* and wild-type mice at 24 months of age, indicating that there is not a *mdx* specific DCM in 24-month-old mice.

### Th2 cytokines induce arginase activity in *mdx* muscle macrophages

**Figure 2:**
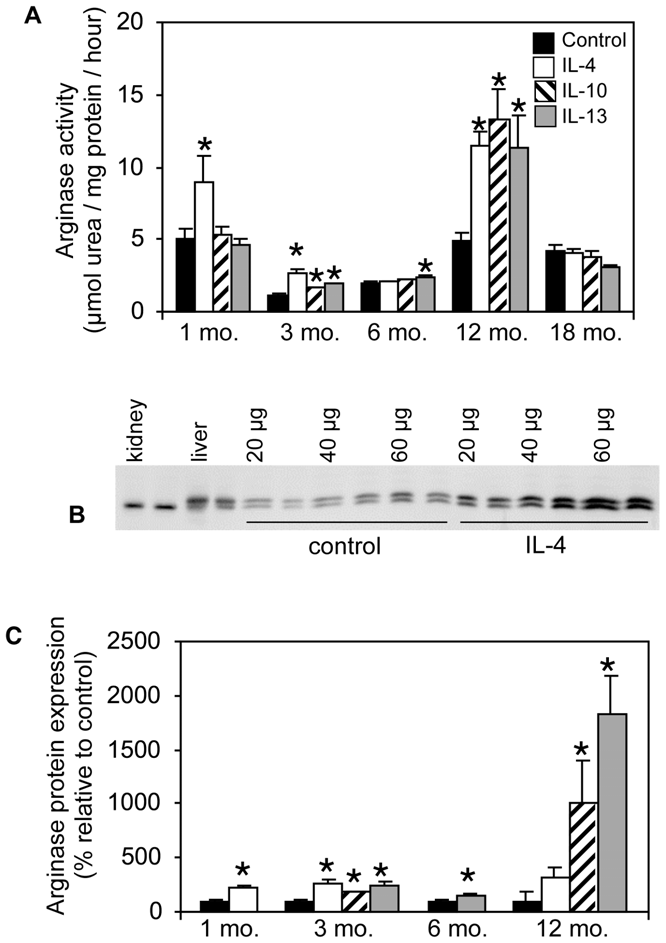
Th2 cytokines induce arginase activity and expression in *mdx* muscle macrophages. (A) Arginase activity of muscle macrophages isolated from *mdx* mice at various ages was measured following in vitro stimulation with either IL-4, IL-10, IL-13 or no cytokine. At least 3 experiments were performed with a minimum of 5 wells for each age and condition. Some error bars are too small to be visible. (B) Representative western blot of 3-month *mdx* muscle macrophage lysates prepared following stimulation with IL-4, or no cytokine, loaded in various quantities as indicated and probed with an arginase-1 and 2 antibody. Homogenates of kidney, which expresses arginase-2, and liver, which expresses arginase-1, were included to show that the antibody recognizes both arginase isoforms. (C) Cytokines that induced increases in arginase activity increased arginase expression. Stimulated and control *mdx* muscle macrophages were analyzed by western blotting as in (B) and densitometrically quantified. * indicates statistical significance at p<0.05 as compared to age-matched control.

Because neither the loss or mislocalization of nNOS was sufficient to cause muscle and cardiac fibrosis or cause defects in cardiac function that resemble DCM, we assayed whether these features of the dystrophic pathology resulted from inflammatory cell mediated processes. We focused on M2 macrophages because they can drive fibrosis through arginine metabolism by arginase, and M2 macrophages have been shown to be present in *mdx* muscle [19], [53]. Furthermore, previous investigations have shown that as the *mdx* pathology progresses, there is an increase in expression of Th2 cytokines that induce an M2 macrophage phenotype, especially IL-4 and IL-10 [19]. Immunohistochemistry confirmed that inflammatory lesions in *mdx* mice contained high concentrations of inflammatory cells that expressed arginase (Fig. 1C). We next tested whether muscle macrophages isolated from *mdx* mice during various stages of the pathology respond to Th2 stimuli by increasing arginase activity and expression. Our findings show that IL-4 and IL-10 each significantly increase arginase activity in *mdx* muscle macrophages, although the magnitude of induction of arginase activity varied with the stage of pathology when the macrophages were collected and with the cytokine used for their stimulation. The strongest responses to Th2-cytokine stimulation were observed in macrophages isolated from 12 month-old *mdx* mice, indicating that M2 macrophages expressing receptors for IL-4, IL-10 and IL-13 are most prevalent at this stage of the disease that temporally coincides with the development of pathological fibrosis in skeletal and cardiac muscle (Fig. 2A). Although each Th2 cytokine tested induced significant increases in arginase activity at 3 and 12 months of age, only IL-4 elicited an increase in arginase activity at 1 month of age, and only IL-13 increased arginase activity at 6 months of age. Despite the severe fibrosis observed in 18 month-old *mdx* mice [present study], Th2 cytokine-stimulation of muscle macrophages isolated from *mdx* mice at the same age did not elicit an increase in arginase activity. We also found that the Th2-induced increases in arginase activity were associated with significantly increased expression of arginase protein by *mdx* muscle macrophages (Fig. 2B and C). Similar to the trend in arginase activity, the greatest increase in arginase expression was observed at the 12-month age-point.

### Deleting arginase-2 expression reduces skeletal muscle fibrosis and kyphosis in *mdx* mice

**Figure 3:**
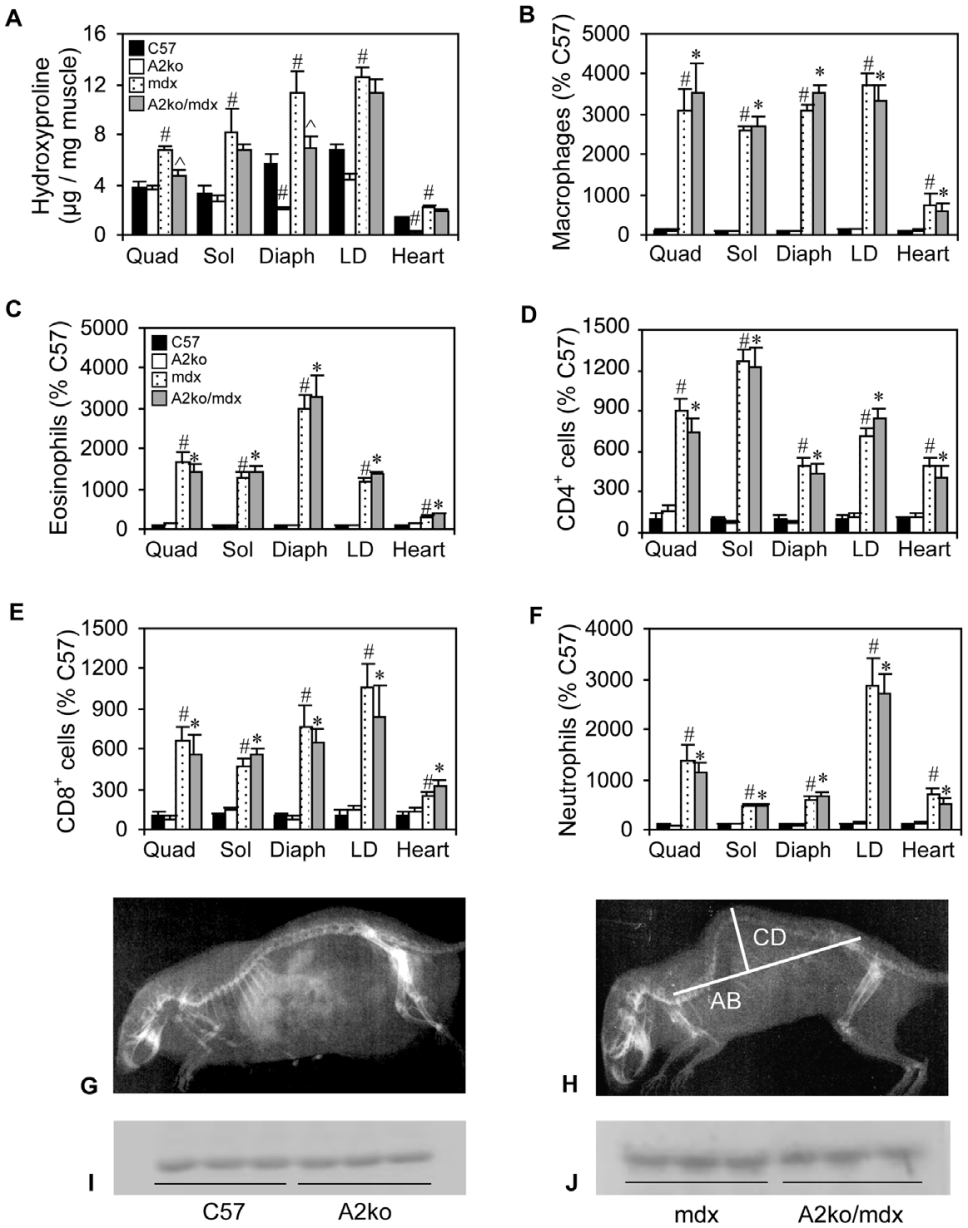
Arginase-2 mutation reduces muscle fibrosis. (A) *Mdx* mice lacking arginase-2 expression demonstrated decreased fibrosis in quadriceps (Quad) and diaphragm (Diaph) muscles. Arginase-2 mutation reduced fibrosis in diaphragm, longissimus dorsi (LD) and heart of wild-type mice. Sol  =  soleus. n = 5 animals per group. (B-F) The concentration of macrophages, eosinophils, CD4+ cells, CD8+ cells and neutrophils in muscles were not affected by arginase-2 mutation. # indicates statistical significance at p<0.05 as compared to C57; ^ indicates statistical significance at p<0.05 as compared to mdx; * indicates statistical significance at p<0.05 as compared to A2ko/wt. n = 7 animals per group. (In A – F, some error bars are too small to be visible.) (G and H) Representative autoradiographs of C57 (G) and *mdx* (H) mice used to measure the kyphotic index (KI). The lines, AB and CD, used to calculate KI are indicated on (H). (I and J) Western blots for arginase-1 in wild-type, C57 mice (I) and *mdx* mice (J) show that null mutation of arginase-2 did not affect arginase-1 expression in muscles from either mouse line. All mice were 18-months old.

**Figure 4:**
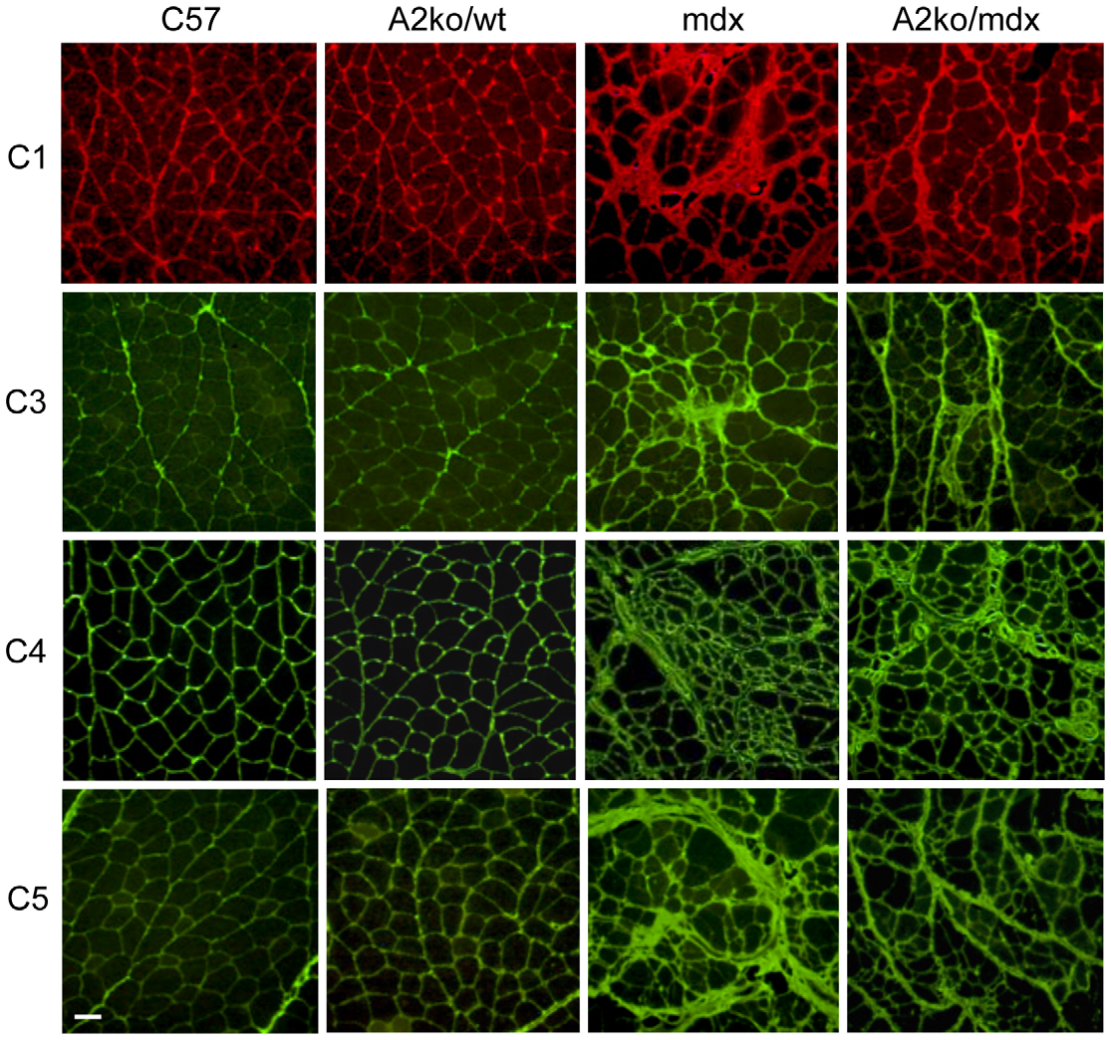
Absence of arginase-2 expression reduces collagen deposition. Cross-sections of quadriceps from C57, arginase-2 null mice on a wild-type background (A2ko/wt), *mdx* and arginase-2 null mice on an *mdx* background (A2ko/*mdx*) were stained with antibodies specific for collagen type I (C1), collagen type 3 (C3), collagen type 4 (C4) and collagen type 5 (C5). No pathological fibrosis is evident in C57 or A2ko/wt quadriceps. *Mdx* mice develop fibrotic lesions and thickening of connective tissue which are reduced in A2ko/*mdx* mice. Bar = 50 µm. All mice were 18-months old.

Because arginine metabolism by arginase-2 in M2 macrophages can drive fibrosis following tissue injury [54], [55] and arginase-2-expressing M2 macrophages are present in elevated numbers in *mdx* skeletal muscle and hearts, we tested whether ablation of arginase-2 in *mdx* mice could affect fibrosis and associated features of dystrophinopathy. Unfortunately, use of a double, arginase-1/-2 knockout mouse for this study was not possible because null-mutation of the arginase-1 gene is lethal by 14 days of age [56]. Dystrophin-deficient mice lacking arginase-2 expression (A2ko/*mdx*) developed significantly less fibrosis in quadriceps and diaphragm muscles than *mdx* mice expressing arginase-2 (Fig. 3A and 4). We also observed a consistent trend toward reduced fibrosis in soleus, longissimus dorsi and cardiac muscle of A2ko/*mdx* mice as well, although these values did not reach statistical significance. Interestingly, arginase-2-null mutation also affected hydroxyproline concentration in wild-type mice (A2ko/wt). The connective tissue content of diaphragms and hearts of A2ko/wt mice was significantly reduced compared to C57 controls. The reduced connective tissue in the A2ko lines was not due to decreased inflammatory cell infiltration since A2ko/*mdx* and A2ko/wt mice showed similar concentrations of macrophages, neutrophils, eosinophils, CD4+ and CD8+ cells (Fig. 3B-F), compared to their arginase-expressing controls in all muscles examined. Kyphosis, which results from fibrosis of postural muscles and significantly contributes to reduced respiratory function in DMD patients [4]–[6], was significantly reduced in A2ko/*mdx* mice (KI: A2ko/*mdx* = 3.4 (sem = 0.11), n = 6; *mdx* =  2.9 (sem = 0.04), n = 10). The absence of arginase-2 had no effect on kyphosis in wild-type mice (KI: A2ko/wt = 4.0 (sem = 0.28), n = 5; C57 = 4.0 (sem = 0.11), n = 7). Null mutation of arginase-2 did not affect arginase-1 expression in either wild-type or mdx muscles (Figs. 3I and 3J).

**Figure 5:**
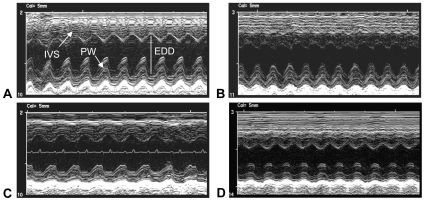
Echocardiography shows that ablation of arginase-2 expression does not affect posterior wall thickness in the left ventricle of *mdx* or wild-type mice. Image taken at the chordal level in the left ventricle shows relative motion of the interventricular septum (IVS) and posterior wall (PW) that delineate the end diastolic diameter (EDD) over time (left to right in image). Representative echocardiographs from (A) *mdx* (n = 5), (B) C57 (n = 7), (C) A2ko (n = 5) and (D) A2ko/*mdx* (n = 6) mice are shown. All mice were 18-months old.

Echocardiography showed that the posterior wall thickness (PWT) of *mdx* mice did not differ significantly from wild-type mice at 18 months (Table 1, Fig. 5). Because the *mdx* hearts are severely fibrotic, this finding indicates that the increase in fibrous tissue in the posterior wall of the left ventricle of the *mdx* heart is similar in magnitude to the loss of contractile tissue in the posterior wall. However, ablation of arginase-2 expression in *mdx* mice did not affect cardiac fibrosis (Fig. 3A) showing that arginase-2 is not required for fibrosis of *mdx* hearts. The reduction of hydroxyproline concentration in wild-type mice in which arginase-2 expression was ablated compared to wild-type hearts that expressed arginase-2 indicates that normal connective tissue accumulation in the myocardium involves arginine metabolism by arginase-2.

### Long-term arginine supplementation increases fibrosis in dystrophin-deficient mice without affecting inflammation

**Figure 6:**
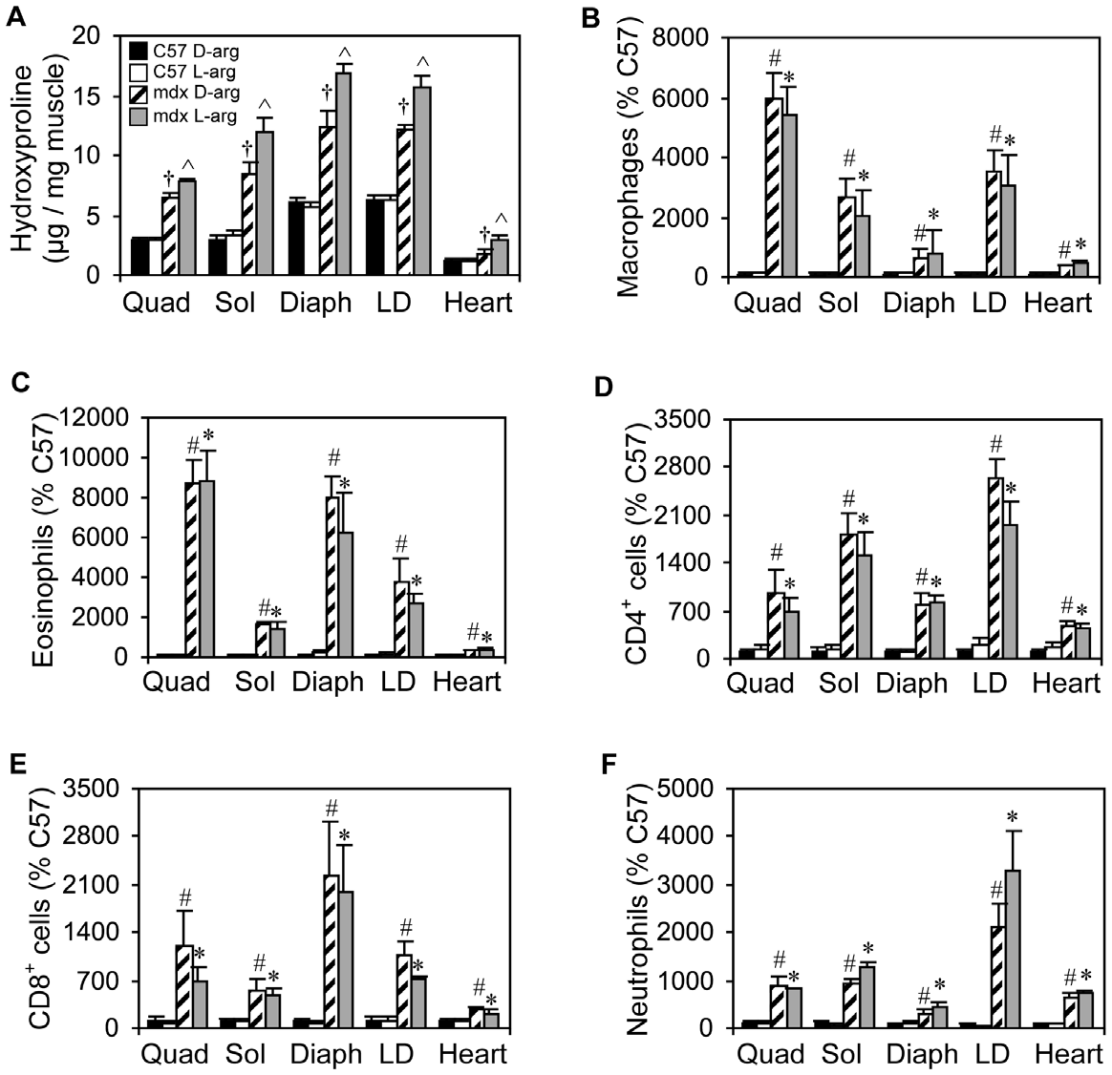
Long-term supplementation with L-arginine increases fibrosis in dystrophin-deficient skeletal muscles and heart, without affecting inflammation. (A) Connective tissue content of all dystrophin-deficient muscles assayed was significantly increased after 18 months of L-arginine treatment. There was no effect on C57 muscles. † indicates statistical significance at p<0.05 as compared to C57 samples. ^ indicates statistical significance at p<0.05 as compared to *mdx* mice treated with D-arginine as well as C57 samples. n = 5 animals per group. (B-F) Long-term treatment with L-arginine did not affect the muscle concentrations of macrophages, eosinophils, CD4+ cells, CD8+ cells or neutrophils. # indicates statistical significance at p<0.05 as compared to C57 D-arg samples. * indicates statistical significance at p<0.05 as compared to C57 L-arg samples. Quad  =  quadriceps, Sol  =  soleus, Diaph  =  diaphragm, LD  =  longissimus dorsi. n = 7 animals per group. Some error bars are too small to be visible.

**Figure 7:**
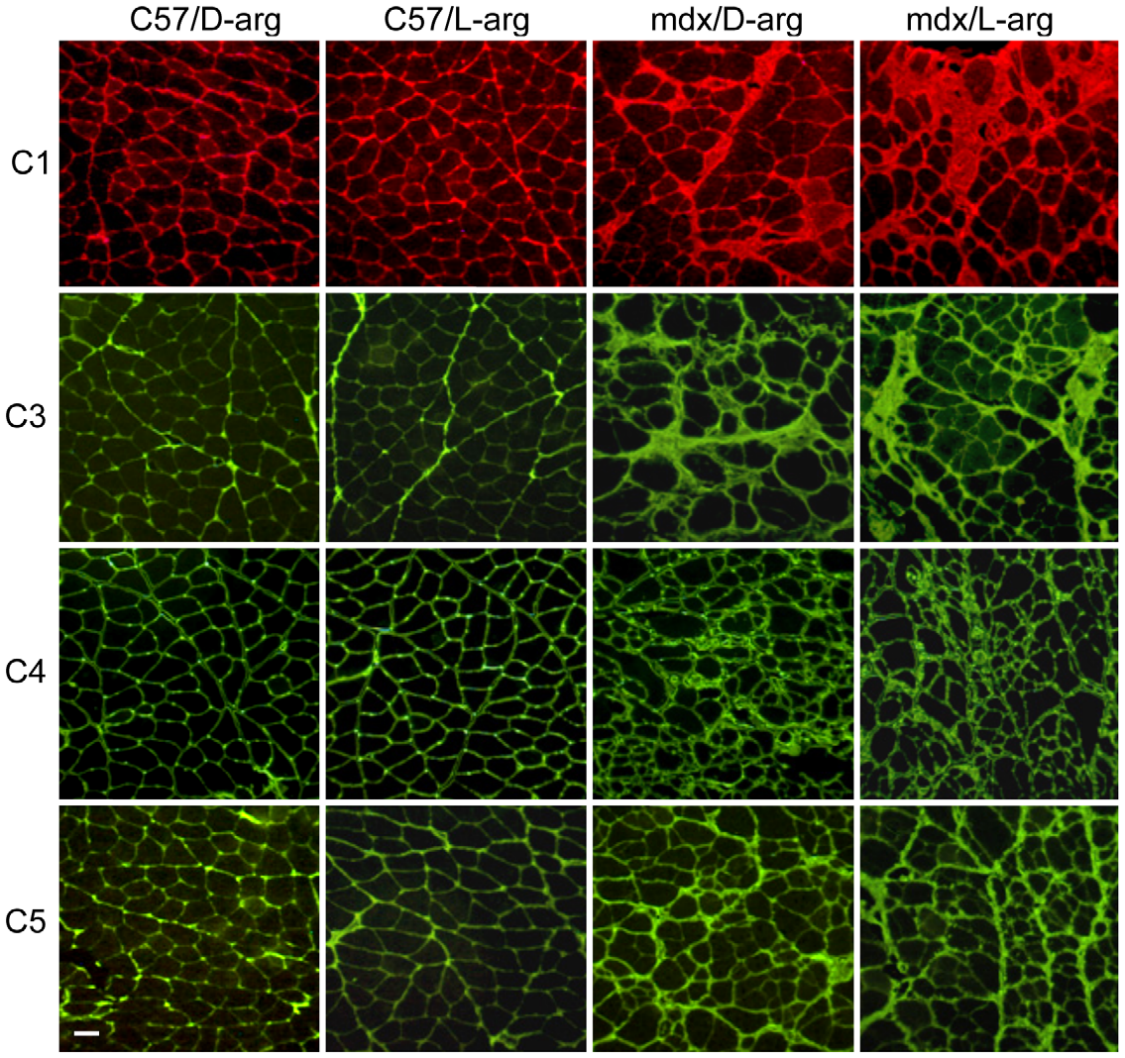
Extended L-arginine treatment increases collagen deposition in *mdx* muscles. Cross-sections of quadriceps isolated from C57 or *mdx* mice treated with L-arginine or D-arginine were labeled with antibodies to collagen type 1 (C1), collagen type 3 (C3), collagen type 4 (C4) and collagen 5 (C5). Collagen distribution is similar in C57 mice treated with D- or L-arginine (C57/D-arg and C57/L-arg, respectively). Significantly more collagen is present in *mdx* mice compared to C57 mice and collagen deposition is more prominent in *mdx* mice treated with L-arginine (*mdx*/L-arg) than *mdx* mice treated with D-arginine (*mdx*/D-arg). Bar = 50 µm. All mice were 18-months old.

The reduced skeletal muscle fibrosis in A2ko/*mdx* mice suggested the possibility that dietary supplementation with arginine could worsen *mdx* muscle fibrosis because arginase activity can be substrate limited. We found that *mdx* mice supplemented with L-arginine from 3 weeks to 18 months of age developed more severe muscle fibrosis than *mdx* mice treated with D-arginine, with increases in connective tissue in quadriceps (+23%), soleus (+43%), diaphragm (+36%) and longissimus dorsi (+30%) observed (Figs. 6A and 7). The pro-fibrotic effect of L-arginine was specific to dystrophin-deficient tissue, as treatment did not induce fibrosis in C57 mice. Muscles from C57 and *mdx* mice treated with D-arginine exhibited connective tissue concentrations similar to untreated mice verifying that the inactive isomer had no effect on fibrosis.

Dietary supplementation with arginine in *mdx* mice also caused large increases in cardiac fibrosis (Fig. 6A). Since fibrosis is the basis of most functional cardiac defects in dystrophin-deficient dystrophy, we examined whether L-arginine treatment affected functional indices measurable by echocardiography. We were not able to detect functional changes in FS, mean circumferential fiber shortening rate (Vcf) or left ventricular ejection fraction (LvEF) in *mdx* mice treated with L-arginine as compared to those treated with D-arginine (Table 2). However, ventricular septal thickness (VST) and PWT were significantly increased in the L-arginine-treated *mdx* mice, compared to *mdx* mice treated with D-arginine. These findings are consistent with our data showing that L-arginine induced an increase in *mdx* cardiac fibrosis. We detected a deficit in *mdx* cardiac function evidenced by decreased FS and LvEF in *mdx* mice treated with L-arginine compared to C57 mice treated with L-arginine (Table 2). Treatment with L-arginine did not have any effect on echocardiographs from C57 mice.

**Table 2:** Echocardiographic analysis of C57 and mdx mice treated with L- or D-arginine.

	VST (mm)	EDD (mm)	PWT (mm)	FS (%)	Vcf (mm/s)	LvEF (%)
C57/D-arg	0.71 (0.02)	4.22 (0.13)	0.70 (0.03)	25.7 (1.61)	5.15 (0.28)	56.3 (2.43)
C57/L-arg	0.77 (0.03)	4.19 (0.11)	0.74 (0.02)	28.2 (1.48)	5.33 (0.33)	60.4 (2.05)
mdx/D-arg	0.67 (0.03)	4.15 (0.12)	0.65 (0.02)	23.1 (1.49)	4.50 (0.34)	52.2 (2.34)
mdx/L-arg	0.79 (0.06)*	4.49 (0.11)	0.75 (0.04)*	22.5 (1.43)^	4.50 (0.31)	51.5 (2.79)^

Values are means with standard errors. VST  =  ventricular septal thickness; EDD  =  end diastolic diameter; PWT  =

posterior wall thickness; FS  =  fractional shortening; Vcf  =  mean circumferential shortening rate; LvEF  =  left

ventricular ejection fraction. Sample sizes: C57/D-arg, n = 5; C57/L-arg, n = 8; *mdx*/D-arg, n = 6; *mdx*/L-arg, n = 7.

* =  statistically significant compared to *mdx*/D-arg at p<0.05. ^  =  statistically significant compared to C57/L-arg at

p<0.05.

We also measured the concentrations of specific inflammatory cell populations in arginine-treated *mdx* mice to test if L-arginine induced *mdx* fibrosis by increasing inflammatory cell numbers. As expected, *mdx* skeletal muscle and cardiac muscle exhibited more inflammation than C57 tissues, including increases in macrophages, eosinophils, neutrophils, CD4+ T cells and CD8+ T cells (Fig. 6B-F). However, immunohistochemical data showed that L-arginine treatment did not affect the concentration of any leukocyte population measured (Fig. 6B-F), suggesting that the increase in fibrosis was not caused by enhancement of inflammatory cell recruitment.

### *Mdx* mice subjected to long-term arginine treatment do not display the benefits reported with short-term arginine treatment

**Figure 8:**
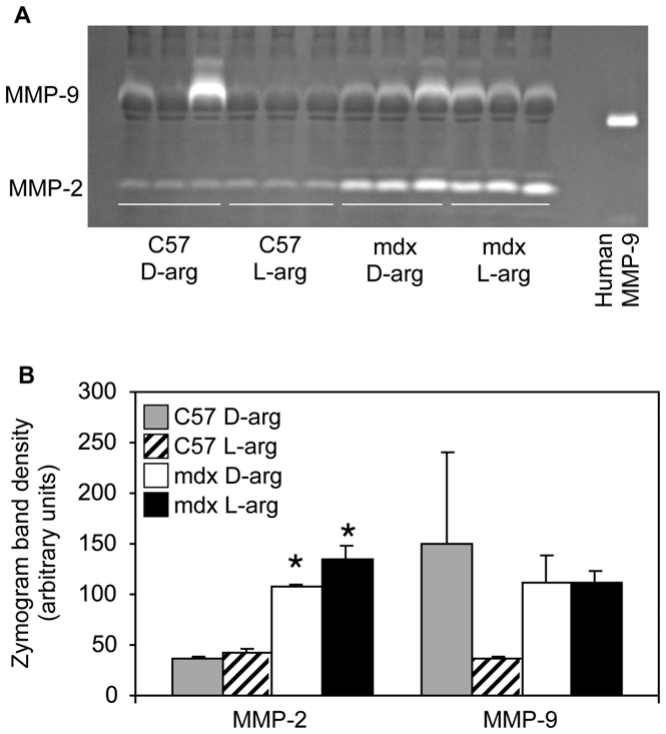
Matrix metalloproteinases 2 and 9 were unaffected following L-arginine treatment. (A) Gelatinase zymogram showing MMP-2 and –9 in muscle homogenates from long-term, L- or D-arginine-treated *mdx* or C57 mice. MMP-9 activity was detected at 100 kDa and MMP-2 activity was detected at 60 and 66 kDa. Human MMP-9 was used as a standard and migrates lower than mouse MMP-9 (47). (B) Densitometric analysis of cleared bands shows that MMP activity is greater in *mdx* tissues, but is not affected by L-arginine supplementation. * indicates significant difference at p<0.05 as compared to treatment-matched C57 samples. All mice were 18-months old.

Previously published results indicate that short-term L-arginine treatment has beneficial effects in *mdx* mice. Hnia et al [33] reported that muscle macrophage number and cytokine expression were decreased after young *mdx* mice were treated with L-arginine for 2 weeks. In the same study, the investigators report that short-term L-arginine treatment decreased the activity of matrix metalloproteinases (MMPs) that is thought to correlate with disease status in *mdx* mice [57]. We tested whether similar effects were associated with our long-term arginine-treatment. First, L-arginine did not affect inflammatory cell infiltration; muscle and hearts from *mdx* mice treated with L-arginine contained concentrations of immune cells that were not different from *mdx* mice treated with D-arginine (Fig. 6B-F). We also measured matrix MMP levels in our long-term L-and D-arginine-treated mice using gelatinase zymography. The levels of MMP in *mdx* and C57 mice were not affected by L-arginine treatment (Fig. 8). MMP-9 was elevated in all *mdx* samples, in agreement with Kherif et al. [44]. While MMP-9 was not detected in the majority of the C57 mice, two samples exhibited high levels that were apparently not related to our experimental perturbation. MMP-2 levels were similarly unaffected by L-arginine treatment in both the *mdx* and C57 groups. MMP-2 was constitutively expressed in C57 and *mdx* mice with greater concentrations in *mdx* muscles, as expected [44].

**Figure 9:**
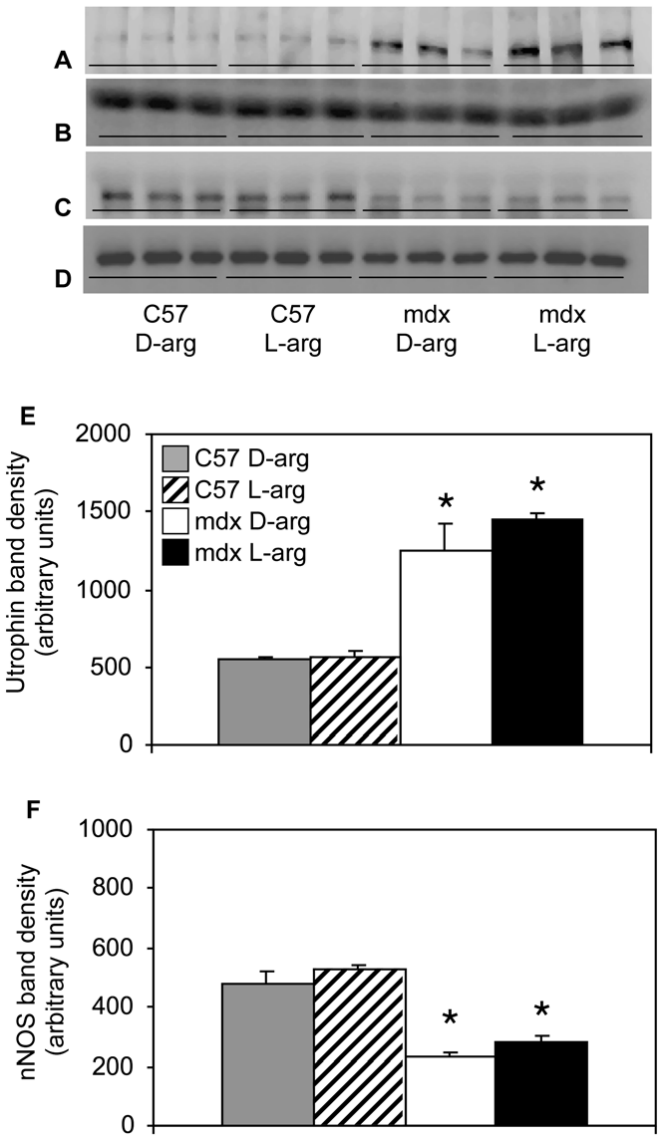
L-arginine treatment does not affect utrophin or nNOS expression. Western blot of tibialis anterior muscle homogenates from long-term, L- or D-arginine-treated *mdx* or C57 mice probed for utrophin (A) or nNOS (C). Blots A and C were stripped and reprobed with anti-skeletal muscle alpha-actin (B and D, respectively) to verify loading consistency. Densitometry of protein expression assessed by western blotting shows that utrophin concentration is greater in *mdx* muscles but is unaffected by L-arginine treatment (E) and that nNOS concentration is greater in wild-type muscles but is unaffected by L-arginine treatment (F). * indicates statistical significance at p<0.05 as compared to treatment-matched C57 samples. All mice were 18-months old.

Several published reports indicate that short-term L-arginine treatment of *mdx* or C57 mice for periods of 2 to 6 weeks can increase expression of the dystrophin homologue, utrophin [31]–[34]. Our analysis of *mdx* and C57 mice shows that 18 months of L-arginine treatment did not significantly affect the expression of utrophin or nNOS, although variability in utrophin concentration was observed in muscles from both L-arginine and D-arginine treated mice (Fig. 9). Collectively, these results indicate that previously reported benefits derived from short-term arginine treatment of *mdx* mice do not persist with extended treatment.

## Discussion

**Figure 10:**
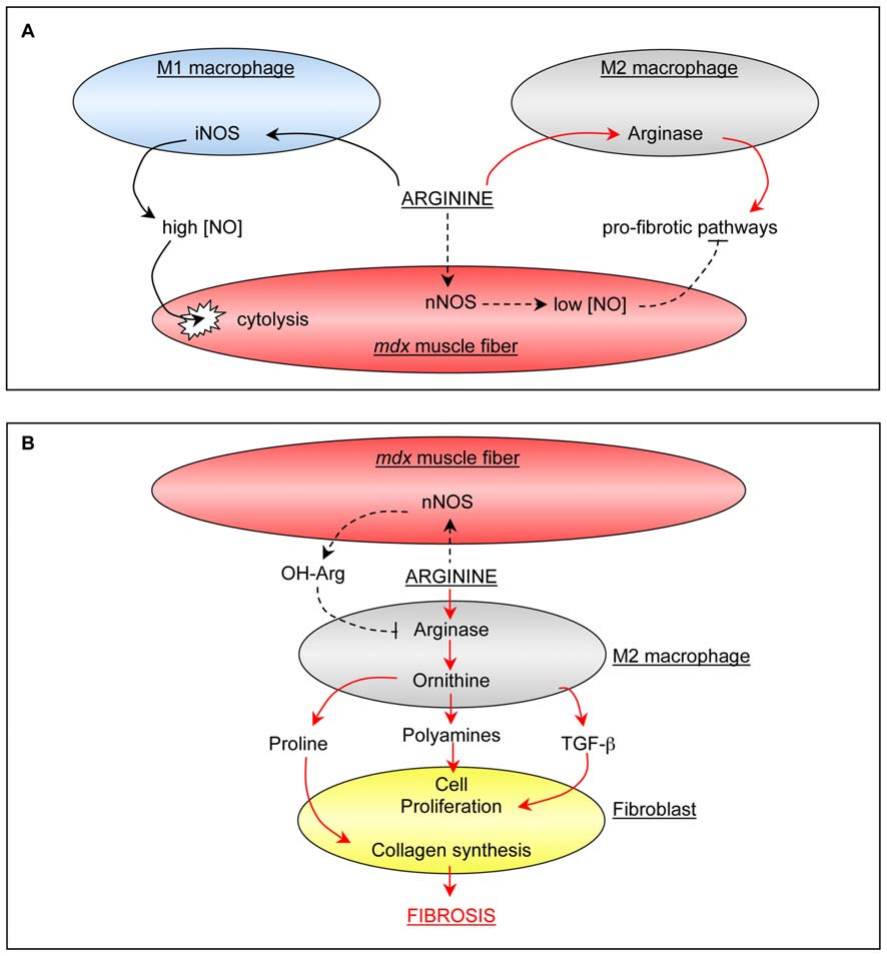
Model of potential, competitive interactions between *mdx* muscle fibers and macrophages for arginine in dystrophic muscles. A: In 4-week muscles, M1 macrophages expressing iNOS and M2 macrophages expressing arginase are present in inflammatory lesions in *mdx* muscle (19). M1 macrophage iNOS and M2 macrophage arginase compete for their common substrate, arginine (19). In wild-type muscle, nNOS in muscle fibers also competes for arginine, but loss of nNOS from dystrophic muscle eliminates that competition, increasing arginine availability for iNOS, increasing cytolysis (19), or arginase, increasing fibrosis (present study). The numbers of iNOS expressing M1 macrophages decline after 4-weeks of age in *mdx* muscle increasing substrate availability for arginase, and the proportion of arginase-expressing macrophages increases by 12-months of age, further driving fibrosis (B). In addition to an increase in arginine for arginase, the loss of several negative regulatory influences of nNOS-derived NO fibrosis occurs. For example, nNOS-derived hydroxyarginine which can inhibit arginase would be diminished. Red arrows  =  profibrotic pathways. Black arrow  =  pathways that compete with fibrotic pathways. Broken arrow  =  pathways that are deficient in *mdx* muscle.

Our findings show that arginine metabolism contributes significantly to fibrosis of dystrophin-deficient muscle. Because the only detectible arginase expression in *mdx* muscle occurs in inflammatory cells, these observations provide insights into a previously unexplored mechanism through which fibrosis can be promoted by the immune system in muscular dystrophy. Our data show that Th2 cytokines, which are expressed at increasingly elevated levels as the *mdx* pathology proceeds [19], increase the expression and activity of arginase in M2 macrophages in *mdx* muscle. We also show that ablation of arginase-2 expression in *mdx* mice reduces fibrosis and decreases kyphosis that may result from fibrosis of paraspinal muscles. Furthermore, dietary supplementation with arginine, which can increase arginase activity, increases fibrosis of skeletal and cardiac muscles in *mdx* mice. Collectively, these data support a model in which perturbations in arginine metabolism promote tissue fibrosis driven by a Th2 inflammatory response that is dominated by M2 macrophages (Fig. 10).

Although our findings show that arginine metabolism by arginase generally increased muscle fibrosis, the relative magnitudes of the effect of arginase-2 mutation on skeletal muscle hydroxyproline content in *mdx* and wild-type muscles varied greatly between the muscles analyzed. For example, the arginase-2 mutation reduced hydroxyproline in *mdx* quadriceps muscles, but not wild-type quadriceps. In the diaphragm, the mutation reduced hydroxyproline in both *mdx* and wild-type but did not affect hydroxyproline concentration in the soleus of either *mdx* or wild-type. These findings reveal new, muscle-specific differences in the pathophysiology of muscular dystrophy. Previous investigators have shown tremendous differences in the course of pathology between different muscle groups in *mdx* mice, with extraocular muscle suffering no pathology, triceps brachii exhibiting more pathology than other limb muscles and diaphragm muscle experiencing the most severe pathology [58], [59]. Our findings add to these differences by illustrating distinctions in the relative importance of arginase-mediated pathways in driving connective tissue production in different muscles, which may underlie a portion of the muscle specific differences in pathology.

Previous studies have identified other mechanisms through which inflammation can promote fibrosis of dystrophin-deficient muscles and hearts. Transforming growth factor-β (TGFβ), a Th2 cytokine that can increase the expression of connective tissue proteins [60]–[65], has been implicated in promoting fibrosis in *mdx* and DMD muscle. However, the pattern of expression of TGFβ in *mdx* and DMD muscle indicates that it may activate fibrosis in early stages of the pathology, but its role is diminished during the later, progressive stages of the disease. For example, TGFβ mRNA is elevated in DMD muscle early in the disease, but then declines while fibrosis continues to progress [66]. Similarly, dystrophin-deficient dogs show a reduction in TGFβ expression in muscle while fibrosis progresses [67] and *mdx* quadriceps muscles show elevations in TGFβ mRNA at early stages of the pathology that then decline to levels that do not differ significantly from wild-type muscles as the disease progresses [68]. However, findings conflict concerning changes in TGFβ expression in the progressively fibrotic diaphragms in *mdx* mice, where fibrosis is an early feature of the disease. Some investigators report an early, transient elevation of TGFβ mRNA in *mdx* diaphragms followed by a rapid decline to control levels [69] while others report a progressive increase in TGFβ mRNA and protein in *mdx* diaphragms over a similar period [53]. Data also suggest that the TGFβ signaling may be most important at the onset of *mdx* pathology; *in vivo* depletions of CD4+ and CD8+ cells from *mdx* mice beginning at 4 weeks of age produced large reductions in circulating TGFβ levels in 24-week old-mice, without reducing diaphragm fibrosis [70].

More recently, major basic protein (MBP), which is released by eosinophils, has been shown to play a major role in promoting fibrosis in dystrophin-deficient muscles and hearts. Eosinophils are also associated with Th2 inflammatory responses and upon activation they can release MBP, which can drive fibrosis [71]. Eosinophils also release IL-4 and IL-10. The elevated production of IL-4 and IL-10 can have multiple effects on driving muscle fibrosis. For example, IL-4 can activate macrophages to a profibrotic, M2 phenotype and IL-10 can deactivate iNOS-expressing M1 macrophages which would increase substrate availability for arginase.

Although our findings confirmed that expression of arginase-2 contributes significantly to muscle fibrosis in *mdx* dystrophy, ablation of arginase-2 expression did not reduce *mdx* cardiac fibrosis. This negative finding was unexpected because previous work has shown that arginase and NOS compete for substrate in the heart, which apparently affects fibrosis. For example, inhibition of arginase in isolated cardiac myocyte preparations increases NO production [72], [73] and elevated expression of nNOS in the myocardium of *mdx* mice reduces cardiac fibrosis [16]. Because cardiac tissue also expresses arginase-1 [72], [73], the lack of effect of arginase-2 mutation on *mdx* cardiac fibrosis may show that arginine metabolism by arginase-1 is sufficient to drive fibrosis in the *mdx* heart. In contrast, our finding that arginase-2 mutation in wild-type hearts greatly reduced cardiac fibrosis shows that arginine metabolism by arginase-2 is important in driving connective tissue production in healthy hearts.

We were surprised to find no significant differences in EDD, FS or LVEF in 18-month-old *mdx* hearts compared to wild-type hearts because a previous study found greater EDD and reduced FS in 10-month-old *mdx* hearts [47]. However, no difference in EDD or FS were observed between *mdx* and C57 hearts at 24 months of age in a subsequent investigation [52], similar to our findings on 18-month-old mice. We attribute the lack of differences between *mdx* and C57 hearts in the old mice (18 to 24 months age) to the occurrence of age-related changes that overwhelm changes that are attributable to the dystrophinopathy and that are apparent in 10-month old mice. Findings by others support this interpretation. For example, echocardiographic data show that FS in wild-type mice decreases significantly between 6 and 18 months of age [74]. A similar trend for decreased FS in wild-type hearts by the age of 16 months has been reported, although the decrease did not reach statistical significance [75]. Significant reductions in the FS of rat hearts have also been reported by 22 months of age in echocardiographic studies [76], that were accompanied by significant increases in EDD.

Perhaps the most striking discovery of this study is that long-term, dietary supplementation with L-arginine exacerbates fibrosis in dystrophin-deficient muscle and heart. Treating *mdx* mice with L-arginine from 3 weeks to 18 months of age induced significant increases in connective tissue in cardiac tissue and all muscles examined. Echocardiography showed that *mdx* mice treated with L-arginine had significantly thicker heart walls which is consistent with increased cardiac fibrosis. These findings are clinically relevant because DMD patients frequently use arginine supplementation in their diets; long-term supplementation could aggravate potentially-lethal features of the pathology including respiratory function and cardiac function as well as spinal deformity and contractures. Nevertheless, there may be benefits to small increases in connective tissue content in dystrophic muscle, if the increases reduced fragility of the muscle cell membrane without causing pathological fibrosis. For example, systemic administration of laminin-111 to *mdx* mice results in deposition of the protein in the endomysium and reduces muscle membrane damage [77].

Our findings also show that the reported, beneficial effects of short-term L-arginine treatment on young, *mdx* mice are not sustained with long-term treatment. Several groups [31]–[34] reported that treating young, *mdx* and C57 mice with L-arginine for 2 to 6 weeks increased utrophin, a dystrophin homologue that can reduce dystrophic pathology [78], [79]. However, our long-term L-arginine treatment had no significant effect on utrophin expression in *mdx* or C57 mice measured at 18 months of age. Additionally, Hnia et al. [33] reported that MMP-2 and –9 were decreased in 5-week-old mice after 2 weeks of L-arginine treatment, which could reflect a reduction of inflammation of *mdx* muscle because immune cells produce MMP [80], [81]. More specifically, reductions in MMP-9 could reflect fewer M1 macrophages because MMP-9 co-localizes with M1 macrophages in *mdx* muscle and MMP-9 mutation reduces macrophages in *mdx* muscle [82]. However, we found that long-term L-arginine treatment had no effect on MMP-2 or MMP-9 levels in *mdx* or C57 mice which may reflect the 18-month treatment period, in contrast to the previous investigation in which the *mdx* mice were treated for 2 weeks early in the disease [33].

In this study, we demonstrate the contribution of arginine metabolism by M2 macrophages to the development of dystrophic fibrosis. We also provide clinically-relevant evidence showing the detrimental effect of long-term L-arginine supplementation on the progression of fibrosis in dystrophic muscle. In the absence of a cure or well-tolerated treatment for DMD, modifying the secondary features of the pathology could lead to significant improvements in health and longevity. Ultimately, we expect that therapeutically modulating the function of specific populations of inflammatory cells during distinct phases of the disease will ameliorate specific features of the pathology.
